# A novel approach to treatment of hypertension in diabetic patients – a multicenter, double-blind, randomized study comparing the efficacy of combination therapy of Eprosartan versus Ramipril with low-dose Hydrochlorothiazide and Moxonidine on blood pressure levels in patients with hypertension and associated diabetes mellitus type 2 – rationale and design [ISRCTN55725285]

**DOI:** 10.1186/1468-6708-5-9

**Published:** 2004-10-01

**Authors:** Cornel Pater, Deepak Bhatnagar, Jean-Pascal Berrou, Joachim Luszick, Katrin Beckmann

**Affiliations:** 1 Department of Cardiovascular Clinical Development, Solvay Pharmaceuticals GmbH, Hannover, Germany; 2 The Royal Oldham Hospital/Manchester Royal Infirmary, UK; 3 Global Product Strategy Department, Solvay Pharmaceuticals GmbH, Hannover Germany

## Abstract

Hypertension and diabetes mellitus are closely interrelated and coexist in as many as two-thirds of patients with type 2 diabetes. The consequent risk of such an association is an accelerated development of atherosclerotic cardiovascular disease and nephropathy complications.

In choosing an antihypertensive agent, effectiveness needs to be accompanied by favourable metabolic, cardioprotective, and nephroprotective properties. Given the multifactorial nature of hypertension, the approach that has gained widespread agreement is treatment with more than one agent. Agents with different mechanisms of action increase antihypertensive efficacy because of synergistic impacts on the cardiovascular system. Combination therapy allows the use of lower doses of each antihypertensive agent which accounts for the excellent tolerability of combination products.

The aim of the present study is to quantify the efficacy of combination therapy of Eprosartan 600 mg respectively Ramipril 5 mg with low-dose Hydrochlorothiazide and Moxonidine on blood pressure levels in patients with essential hypertension and associated diabetes mellitus type 2.

The use of monotherapy (Eprosartan or Ramipril) followed by addition of low-dose Hydrochlorothiazide as second agent and of Moxonidine as a third agent will be individualized to the severity of hypertension in the particular patient and to his/her degree of response to current treatment.

## Background

The clinical combination of hypertension and diabetes carries a particular poor prognosis [1-6]. Clinical studies done in individuals with type 2 diabetes and substudies obtained from clinical trials done in the general population have demonstrated that achievement of *goal *blood pressure (< 130/80 mm Hg) in this patient category is crucial in decreasing the premature morbidity and mortality [7]. Thus, management of subjects with type 2 diabetes and associated hypertension needs to be early and aggressive, and must use a global approach. Findings from large, international outcomes studies as well as guidelines and recommendation of prestigious international scientific bodies have made available consensus recommendations [8-13].

The challenge clinicians are facing is to tighten blood pressure control to less than 130/80 mmHg and to adjust initiation of therapy to the severity of hypertension in the individual patient.

This multicenter study will evaluate the efficacy and tolerability of monotherapy, double- and triple- antihypertensive combination therapies in a large spectrum of hypertension & diabetes patient population, as summarised in Table 1.

**Table 1:** Large spectrum of hypertension and diabetes patient population selected for the multicenter study that will evaluate the efficacy and tolerability of monotherapy and double and triple-antlhy pertensive combination therapies

*Goal*BP*	**Threshold**	**Upper limit**
for all patients regardless BP values	for initiation of double-combination	of BP values targeted
**< 130/80 mmHg**	**> 150/90 mmHg**	**≤ 179/109 mmHg**

* The *Goal *BP defines the cut off point for *responders*/*non-responders *to any therapy.

Table 2 (see Additional file 1) specifies the treatment strategies to be employed in the study as adjusted to severity of hypertension in the particular patient and to his/her degree of *response *to that therapy.

The primary objectives of hypertension management in patients with diabetes are to reduce blood pressure levels to currently recommended target level and thus to reduce the risk of cardiovascular and renal complications without adversely impacting glycemic and lipid control.

Previous debate regarding the level of blood pressure reduction that optimizes cardiovascular risk reduction is currently settled. BP goal of < 130/85 mmHg promoted by the JNC-VI guidelines issued 1997 [10] were replaced in 2002 by a position paper of the American Diabetes Association (ADA) supporting a target blood pressure in hypertension & diabetes patients of < 130/80 mmHg [14]. This blood pressure-goal is also endorsed by the most recent JNC-7 guidelines [15] and two other American professional societies [16,17] as well as by the ESH/ESC [9] and formally by the ISH.

A widespread agreement, supported by the above mentioned organizations/societies is in place, regarding the principles governing the use of appropriate antihypertensive drug combinations to maximize hypotensive efficacy while minimizing side effects. Polypharmacy is common place and, with at least one third of patients requiring two or more agents simultaneously, a paradigm shift in the approach of initiating therapy is done by advocating use of two agents in subjects with more severe hypertension (BP in excess of 20/10 mmHg above goal). Low-dose thiazide diuretic is favored as one of the two starting agents.

In general, monotherapy is likely to be successful in mild hypertensive patients (grade 1 hypertension) without associated major risk factors for CHD. In contrast, patients with type 2 diabetes need more rigorous control of BP in an easier, simpler fashion, given the remarkable complexity of the multiple drug regimens needed to control their comorbid medical problems (e.g., diabetes, obesity, high cholesterol).

A large body of evidence derived from a multitude of international trials have demonstrated both the benefit of low-level, goal blood pressure, in terms of prevention of long-term complications and, the need for multiple drug combinations in order to achieve that goal [13,18-20]. Furthermore, in a computer-modelled cost-effectiveness analysis of the JNC-VI treatment goal (< 130/85 mmHg), lowering blood pressure to goal increases patients' life expectancy and decreases long-term cost [21]. Cost-effectiveness analysis in the context of the UKPDS study has also revealed that incremental cost of tight control (< 150.85 mmHg) versus less tight control (< 180/105 mmHg) was considered to be effective [22].

In the HOT study [13], which recruited grade 2 and 3 hypertensives after washout from previous agents, monotherapy was successful in only 25–40% of patients, according to the target diastolic blood pressure. In trials of diabetic patients, the vast majority were on at least two drugs, and, in two recent trials on diabetic nephropathy [23,24] an average of 2.5 to 3.0 non-study drugs were required in addition to the angiotensin receptor antagonist used in these studies (losartan/irbesartan).

Given the very poor BP control rate, i.e., 11% in patients with hypertension & diabetes, the use of combination therapy is an important therapeutic consideration, as it facilitates quicker and easier attainment of goal BP and should lead to a greater proportion of people with diabetes who achieve BP goal. Initiation of treatment by combination therapy was effectively tested in the VA study at the beginning of the antihypertensive treatment trial era [25,26] and recently in the PROGRESS study [27].

## Methods

### Patient Population

Subjects will be recruited in outpatient clinics/offices of general practitioners and internal medicine/cardiology specialists from the entire spectrum of patients having coexistent hypertension and diabetes mellitus type 2. The upper limit of blood pressure values targeted (≤ 179/109 mmHg) correspond grade 2 hypertension (according to current ESC/ISH and WHO guidelines).

Subjects to be recruited are supposed to have both entities (hypertension with BP values in the range ≥ 130/80 – ≤ 179/109 mmHg and diabetes mellitus type 2) diagnosed since previously, undergoing current antihypertensive treatment and, to be eligible for the study according to the specific inclusion/exclusion criteria described below.

The study consists of five distinct phases: Screening (S) (up to seven days), Placebo Run-in phase (two weeks), monotherapy (four weeks, double-blind fashion), double-combination treatment (Eprosartan/HCTZ respectively Ramipril/HCTZ for four weeks, HCTZ open labelled), triple-combination treatment (Eprosartan/HCTZ/Moxonidine versus Ramipril/HCTZ/Moxonidine with HCTZ and Moxonidine open labelled) and Follow-up (two to seven days). Patients allocated to monotherapy will participate in the study for a four weeks period while those starting with double combination therapy will receive medication for a maximum of 12 weeks.

**Figure 1:**
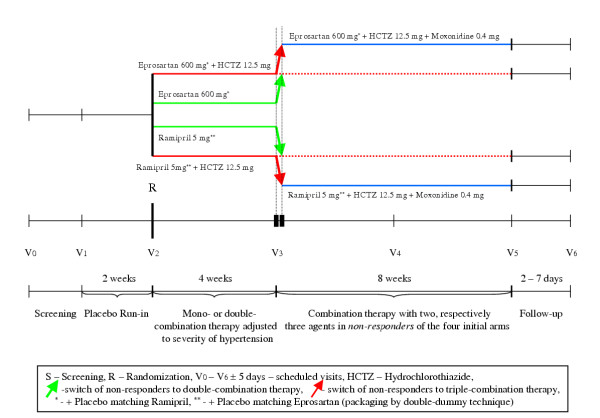
Study Design. A multicenter, double-blind, randomized study comparing the efficacy of combination therapy of Eprosartan versus Ramipril with low-dose Hydrochlorothiazide and Moxonidine on blood pressure levels in patients with essential hypertension and associated diabetes mellitus type 2.

The flowchart below captures the main events during the study conduct (Fig. 1).

### Randomization and Blinding

Randomization will be concealed.

A stratified randomization will be employed based on the following rules:

1. Subjects with blood pressure in range: BP ≥ 130/80 – ≤ 150/90 mm Hg will be randomly allocated to one of the monotherapy arms (Eprosartan or Ramipril). 30 subjects will be recruited for each arm.

2. Subjects with blood pressure in the range: BP > 150/90 – ≤ 179/109 mm Hg will be randomly allocated to double-combination therapy (Eprosartan/HCTZ or Ramipril/HCTZ); 190 subjects will be recruited for each arm.

The randomization lists will be provided by the Department of Clinical Supplies at Solvay Pharmaceuticals BV with the program ADLS. Patients will be allocated in equal numbers to each sequence. A fixed block size of patients will be used, and only complete blocks of study medication will be provided to the centers.

Within each center, randomization numbers will be used in ascending order and patients will be allocated to randomization code numbers in chronological order. The study will be unblinded when all CRFs are in house and the data on the database have been declared clean.

The following drugs are to be used in the study:

• Eprosartan 600 mg, once daily

• Ramipril 5 mg, once daily

• Hydrochlorothiazide 12.5 mg, once daily

• Moxonidine 0.4 mg, once daily

• Placebo tablets matching Eprosartan 600 mg and Ramipril 5 mg will be used in the monotherapy, in the double- and in the triple-combination therapy phases. Hydrochlorothiazide tablets will be open labelled in the double- and triple-combination therapy phases. Moxonidine tablets will open-labelled in the triple-combination therapy phase.

**Figure 2:**
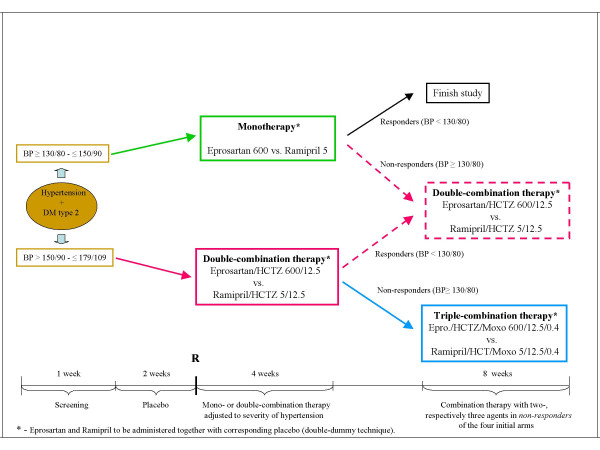
Treatment Algorithm

Eprosartan and Ramipril and the corresponding placebos will be packaged according to the double-dummy technique. Fig. 2 summarizes the treatment algorithm.

### Monotherapy (Eprosartan vs. Ramipril)

Patients eligible for participation in the study by the assessment at V2 will be randomized on the basis of the severity of their hypertension and allocated to either monotherapy or to double-combination therapy (described below).

Patients with initial blood pressure values in the range ≥ 130/80 – ≤ 150/90 mmHg, will be randomly allocated to one of the monotherapy groups.

By the end of the first four-week phase an assessment will be made as to whether patients have reached or not the "goal" hypertension (< 130/80 mmHg).

Patients on monotherapy deemed to be responders at the end of four weeks treatment (i.e., have reached the "goal") will terminate their participation in the study and be followed up during the ensuing two to seven days after stopped monotherapy (procedure similar with Follow-up at end of study (V6).

Non-responders (according to above mentioned criteria, i. e., with BP still ≥ 130/80 mmHg), will receive double-combination therapy (Eprosartan/HCTZ respectively Ramipril/HCTZ) and will be followed-up for eight weeks (red-dotted line in the flowchart).

### Double-combination Therapy (Eprosartan/HCTZ vs. Ramipril/HCTZ)

Patients with initial blood pressure values in the range > 150/90 – ≤ 179/109 mmHg, will be randomly allocated to double-combination therapy. By the end of this first four-week phase an assessment will be made as to whether patients have reached or not the "goal" hypertension (< 130/80 mmHg).

*Responders *will maintain double-combination therapy and will be followed-up for an eight week period (V3 toV5, dotted-line in the flowchart) and retain therapy unchanged.

*Non-responders *will receive triple-combination, as described below.

### Triple-combination Therapy (Eprosartan/HCTZ/Moxonidine vs. Ramipril/HCTZ/Moxonidine)

*Non-responder *patients after four weeks of double-combination therapy will receive triple-combination (Eprosartan/HCTZ/Moxonidine vs. Ramipril/HCTZ/Moxonidine) and will be followed-up for an eight weeks period (V3 to V5).

After the first four weeks of monotherapy respectively double-combination therapy (V3), patients will be reassessed for compliance, adverse events and supplied with medication for the next eight weeks (except for the monotherapy patients who reached goal blood pressure and who will terminate the study).

During the eight weeks triple-combination therapy all patients will be reassessed for compliance and adverse events (visits V4, V5 and V6). A 12-lead ECG will be performed by S, V3 and V5 while safety laboratory parameters will be performed by S, V2 and finally by visit V5.

A Follow-up Visit will be performed on all patients with full physical examination, BP and pulse rate check within the ensuing two to seven days after study end (V6). Further follow-up and optimal treatment will be decided on a case-by-case basis by the physician in charge. Table 3 (see Additional file 2) displays a summary of the scheduled investigations, as planned for each particular visit.

### Inclusion Criteria

1. Males and females aged 40 to 80 years of age. Women of childbearing age will be subject to pregnancy testing and will agree to maintain adequate hormonal contraception.

2. Eligible patients should have diagnosed essential hypertension (not controlled with current treatment, i.e., BP ≥ 130/80 – ≤ 179/109 mmHg) and diagnosed associated diabetes mellitus type 2, willing to accept withdrawal of any antihypertensive medication by the time of the Screening visit.

### Exclusion Criteria

A multitude of exclusion criteria, carefully listed in the study protocol, can be summarised in three different groups:

1. Ineligibility based on hypertension grade 3 (BP ≥ 180/110 mmHg), any form of secondary hypertension or hypotension (SBP ≤ 90 mmHg).

2. Any form of organic heart disease requiring medical treatment that might have hypotensive effect, imply need for invasive investigation or surgery.

3. The patient is suffering from a severe concomitant illness related to any body organ or system, likely to affect outcome assessment. Likewise, ineligibility is declared for patient anticipated to have compliance problems, participants in another trial during the past 30 days, pregnancy and lactations and known hypersensitivity to ingredients of any of the employed agents (eprosartan, ramipril, hydrochlorothiazide, moxonidine). In addition, diabetes mellitus type 1 is exclusion criteria.

## Study Outcomes

### Prior and Concomitant Therapy

The study protocol calls for every patient to be treated optimally by the physician in charge and to receive comprehensive, individualized *lifestyle change *advice regarding relevant diet and physical activity. Visits are to be scheduled in the context of the study (at four weeks interval during ongoing treatment). Any antihypertensive medication should be withdrawn latest by Screening visit and will be prohibited during the whole period of ongoing study.

### Ethics and Informed Consent

The study will be conducted in accordance with ICH GCP and the European Directive 2001/20/EC of the European Parliament and of the Council of 4 April 2001 (on the approximation of the laws, regulations and administrative provisions of the member states relating to implementation of good clinical practice in the conduct of clinical trials of medicinal products for human use) and on the basis of ethical principles laid down in the current revision of the Declaration of Helsinki (Edinburgh 2000). In addition, Solvay Pharmaceuticals GmbH policies and procedures should also be followed.

Written consent, involving provision of detailed information regarding the study objectives, design, scope of the intervention, risks and benefits, will be obtained for all patients before initiating any study procedures. Likewise, study documentation is to be subject to the scrutiny of local ethical committees in the two countries participating in the study.

### Sample Size and Statistical Analysis

#### Efficacy

The primary objective is to demonstrate the superiority of combination therapy of Eprosartan/HCTZ (600/12.5 mg) versus Ramipril/HCTZ (5/12.5 mg) with the primary parameter of attention being the percentage of patients brought to *goal *blood pressure (<130/80 mmHg) at visit V3.

Null hypothesis: H_0_: P_E+HCTZ _= P_R+HCTZ_

Alternative hypothesis: H_1_: P_E+HCTZ _≠ P_R+HCTZ_,

Where P_E+HCTZ _is the percentage of patients brought to goal blood pressure at visit 3 with Eprosartan/HCTZ and P_R+HCTZ _is the percentage of patients brought to goal blood pressure at visit V3 with Ramipril/HCTZ.

The primary parameter will be analyzed using the Cochran-Mantel-Haenszel test, controlling for center effects. Statistical significance will be assessed with a two-sided test at 0.05 α level. The confirmative analysis of the primary parameter will be performed on the intent-to-treat patient sample.

Secondary efficacy objectives:

• To compare the mean change in sitting systolic blood pressure (sitSBP) and sitting diastolic blood pressure (sitDBP) between Eprosartan/HCTZ and Ramipril/HCTZ (V3 vs. V2).

• To compare the percentage of patients brought to goal blood pressure by the triple-combination Eprosartan/HCTZ/Moxonidine vs. Ramipril/HCTZ/Moxonidine (V5 vs. V3).

• To compare the mean change in sitSBP and sitDBP between triple-combination with Eprosartan/HCTZ/Moxonidine vs. Ramipril/HCTZ/Moxonidine (V5 vs. V3).

• To compare the mean change in sitSBP and sitDBP between monotherapy with Eprosartan vs. Ramipril (V3 vs. V2)

• To compare the percentage of patients brought to goal blood pressure at visit V5 between Eprosartan/HCTZ and Ramipril/HCTZ in patients not at goal blood pressure after four weeks of monotherapy.

• To compare the mean change in sitSBP and sitDBP as well as the responder rate in patients non-responders (not *at goal*) after four weeks of monotherapy (switched to double combination therapy) (V5 vs.V3); and to compare the mean change in sitSBP and sitDBP as well as the responder rate maintenance in patients who reached *goal *blood pressure value at the end of first four weeks of double-combination therapy and successively entered an eight weeks follow-up period (V5 vs.V3).

Changes in blood pressure parameters will be assessed by analysis of covariance (ANCOVA). The model will include the intercept, treatment and center as fixed effects and the baseline value as covariate. For response rates, the treatment groups will be compared using the Cochran-Mantel-Haenszel test, controlling for center effects. Comparisons of the medication regimens will be reported along with 95% confidence intervals of the relative risk ratios. These analyses will be considered as exploratory.

#### Safety

All patients who receive at least one dose of double-blind medication will be assessed for clinical safety and tolerability. Evaluation of safety data will be based on comparisons of patient experience by treatment group. Clinical interpretation of safety will be based on reviews of standard displays of adverse events incidence, pulse rate data, and laboratory test values. Summary statistics of laboratory test values and incidence of adverse events according to treatment and time of onset will be presented.

### Sample Size, Power and Level of Significance

A formal sample size estimation has been done for patients with blood pressure in the range: > 150/90 mmHg and ≤ 179/109 mmHg.

Assuming that 55% of the patients in the Eprosartan/HCTZ group would reach *goal *blood pressure as compared with only 40% in the Ramipril/HCTZ group, a 0.05% two-sided significance level with 80% power to detect the targeted 15% difference will imply the need for 346 patients supposed to complete the four-weeks double-combination therapy phase. Further 35 patients (10% of the total) will be recruited to account for drop-outs.

In addition, 60 subjects with BP ≥ 130/80 and ≤ 150/90 mmHg will be randomly allocated to either Eprosartan or Ramipril monotherapy group at visit V2. Inclusion of monotherapy phase with a relatively low number of patients (30 subjects per arm) is justified by the intention to therapeutically target the whole spectrum of patient population having coexistent diabetes mellitus type 2 and mild to moderate hypertension, in whom the agents tested are likely to be effective.

### Blood Pressure Measurements

Office blood pressure will be determined by Riva-Rocci method with a mercury or a mercury calibrated sphygmomanometer throughout the study. All measurements will be made on the same arm supported at heart level, using the same cuff size and the same equipment. If the patient's arm circumference is > 32 cm, a large blood pressure cuff should be used. Diastolic blood pressure will be measured at the disappearance of Korotkoff sounds phaseV. Measurements should be taken by the same staff member at the particular visits.

For an individual patient blood pressure measurements should be performed at 24 hours after the last oral dose, at the same time (± 2 hour) in the morning, between 8 and 10 am. Blood pressure will be measured in the following sequence: after the patient sits quietly for at least 5 minutes, blood pressure will be measured twice at approximately 2-minutes interval. The average of these measurements will be recorded. If the difference between measurements is in excess of 5 mmHg a third reading will be performed and the average value recorded as mean sitting systolic and diastolic blood pressure.

Measurements should be performed by the same study assistant using the same device, in each of the centres involved in the study.

## Discussion

The current evidence base is strongly in favour of combining drugs in order to achieve blood pressure goals, in particular in patients with coexistent hypertension & diabetes. Likewise, there is a widespread agreement in the scientific community as to the *goal *blood pressure to be achieved in these patients. Further, common sense in clinical practice dictates that combination therapies should be tailored to severity of hypertension in the individual patient and that, eventual associated risk factors/comorbidities should be accounted for in the process of treatment decision making.

Patients with high blood pressure and associated impaired glucose tolerance or overt diabetes mellitus type 2, as a group, are insulin resistant, [28] glucose intolerant [29-31], hyperinsulinemic [32-36], dyslipidemic [37-42] and with evidence of endothelial dysfunction [43,44]. Extensive epidemiological evidence indicates that diabetic individuals with hypertension have greatly increased risk of cardiovascular disease, renal insufficiency, and diabetic retinopathy [45-47].

For every 5 to 10 mmHg decrease in systolic blood pressure achieved with diuretics, ARBs, ACE inhibitors, beta-blockers or calcium channel blockers in patients with diabetes, there is a 20% to 30% relative risk reduction in cardiovascular events [48-53].

Agents belonging to the nine, most well-known different antihypertensive drug classes produce a similar reduction in systolic and diastolic blood pressure (10–15 and 5–10 mm Hg respectively). Differences in terms of magnitude of blood pressure lowering, as indicated by results from comparative efficacy studies, are usually small [54]. However, larger differences have been shown as to effects on hard endpoints (myocardial infarction, heart failure, stroke).

Comparisons between different agents in patients with hypertension & diabetes mellitus type 2, convincingly point to ACE inhibitors and ARBs as being the two classes of antihypertensive drugs that reduce the activity of the renin-angiotensin II system, and should be among the preferred first-step drugs for the treatment of these conditions [55]. Angiotensin II increases blood pressure by enhancing aldosterone synthesis, resulting in sodium retention and direct vasoconstriction. The first step in this pathway is inhibited by adrenergic blockers. The third and forth steps are inhibited by ACE inhibitors and ARBs, respectively [56].

Clinical trials carried out world-wide have shown that ACE inhibitors have renoprotective effects [57,58] and clear cardiovascular benefits [59-63]. Their main side effects are dry cough and angioedema.

In contrast, in placebo-controlled trials, the ARBs have demonstrated almost no side effects [64]. Both ACE inhibitors and ARBs have been shown to maintain quality of life of hypertensive patients equal to or better than other classes of antihypertensive drugs [65-68]. The only laboratory abnormality that may occur with agents from both classes is mild hyperkaliemia, especially in some elderly patients with type 2 diabetes who have hyporeninemic-hypoaldosteronism [69].

Use of low-dose thiazide diuretic (< 25 mg) as a second agent in treatment of patients with hypertension & diabetes is well-documented and widely recommended [21,70-73]. It has beneficial effects on both morbidity and mortality figures while, previous general concern on the negative impact of diuretics on the different lipid parameters is no longer justified as, all long-term studies with low-dose diuretics have not been shown to affect lipid profiles in a negative way [74-76]. Moreover, in studies of a year or more, diuretics have been shown to reduce cardiovascular risk in every trial to date [77-79].

Since drug combinations may be required for many years in the age-groups in which type 2 diabetes is most prevalent, there have been calls for the use of agents devoid of adverse effects on carbohydrate and lipid metabolism. It has been suggested that such effects may account for the shortfall in reduction of coronary heart disease observed in clinical trials of diuretics and β-blockers [80,81].

Moxonidine stimulates imidazoline-I_1 _receptors in the medulla, thereby reducing central sympathetic drive and attenuating peripheral vascular resistance. In addition, reduced sympathetic drive results in lower plasma concentrations of catecholamines and renin. Randomised comparative studies show that the efficacy of moxonidine as monotherapy is similar to that of other antihypertensive agents [82]. Moreover, selectivity for the I_1 _receptor greatly reduces the adverse affects attributable to costimulation of medullary α_2_-adrenoceptors [82] observed with the first generation of centrally acting agents, α-methyldopa and clonidine.

In clinical studies, moxonidine has been shown to have neutral or beneficial effects on lipid and carbohydrate metabolism [82,83]. A retrospective analysis suggested minor dose-dependent reductions in fasting plasma glucose in moxonidine-treated hypertensive patients.

On the available evidence, moxonidine seems to be a logical choice as component of combination treatment of patients with hypertension and associated diabetes mellitus type 2 or impaired glucose tolerance.

## Conclusions

The poor blood pressure control in patients with hypertension & diabetes in everyday life lies, at least in part, in the emphasis in the evidence-based guidelines of the recent past towards advice on initial, single treatments as well as in their lack of clarity and transparency in recommending pre-specified blood pressure targets [10,84-86].

Previous consensual advice that combination treatments expose patients to the increased risk of adverse events has been replaced by good evidence to the contrary: use of several agents combined or of fixed-dose combinations treatments have the potential to bring patients to *goal blood pressure *and thereby to minimize long term risk of hypertension/diabetes-related complications [22-27,87].

Despite the apparent simplicity of the paradigm shift towards clear *blood pressure goal *and *individualized therapy on the basis of hypertension severity *(and addition of a third agent in case of uncontrolled BP with two agents), comparative data to guide clinical practice is still lacking and this applies also to the comparison of ARBs versus ACE inhibitors. The present study attempts to explore this area.

## Competing interests

Pater C, Berrou JP, Luszick J and Beckman K are employees of Solvay Pharmaceuticals.

## Authors' contributions

Study concept and design: Pater, Berrou, Luszick

Drafting of manuscript: Pater, Bhatnagar

Statistical expertise: Beckman

## Supplementary Material

Additional File 1 Table 2 – Blood pressure-adjusted treatment stratification Click here for file

Additional File 2 Table 3 – Investigations Schedule Click here for file
