# Supplementary material for: A novel approach to treatment of hypertension in diabetic patients – a multicenter, double-blind, randomized study comparing the efficacy of combination therapy of Eprosartan versus Ramipril with low-dose Hydrochlorothiazide and Moxonidine on blood pressure levels in patients with hypertension and associated diabetes mellitus type 2 – rationale and design [ISRCTN55725285]
Source: Curr Control Trials Cardiovasc Med. 2004 Oct 1;5(1):9. doi: 10.1186/1468-6708-5-9 (PMC524514; doi:10.1186/1468-6708-5-9)
Supplement: Additional File 1 — Table 2 – Blood pressure-adjusted treatment stratification [file 1468-6708-5-9-S1.doc]

| Therapeutic BP range* Agent(s)**  Strategy (mmHg) (mg) |
| --- |
| MONOTHERAPY ≥ 130/80 - ≤ 150/90 Eprosartan 600 vs. Ramipril 5  DOUBLE- > 150/90 - ≤ 179/109 Eprosartan/HCTZ vs. Ramipril/HCTZ  COMBINATION + *non-responders* to 600/12.5 5/12.5  monotherapy  TRIPLE- *Non-responders* to Eprosartan/HCTZ/Moxo vs. Ramipril/HCTZ/Moxo  COMBINATION double-combination 600/12.5/0.4 5/12.5/0.4 |

*When a patient’s systolic and diastolic blood pressure fall into different categories the higher category

should apply!

**Eprosartan and Ramipril are administered with corresponding Placebo (double-dummy technique).
