# Supplementary material for: A novel approach to treatment of hypertension in diabetic patients – a multicenter, double-blind, randomized study comparing the efficacy of combination therapy of Eprosartan versus Ramipril with low-dose Hydrochlorothiazide and Moxonidine on blood pressure levels in patients with hypertension and associated diabetes mellitus type 2 – rationale and design [ISRCTN55725285]
Source: Curr Control Trials Cardiovasc Med. 2004 Oct 1;5(1):9. doi: 10.1186/1468-6708-5-9 (PMC524514; doi:10.1186/1468-6708-5-9)
Supplement: Additional File 2 — Table 3 – Investigations Schedule [file 1468-6708-5-9-S2.doc]

| Time Frame  Investigations | | Screen | V1 | V2(R) | V3 | V4 | V5 | V6 |
| --- | --- | --- | --- | --- | --- | --- | --- | --- |
| Informed Consent | x | | | | | | | |
| Incl/Excl Criteria | x x x | | | | | | | |
| Medical History | x | | | | | | | |
| Physical Exam | x x x | | | | | | | |
| BP + Pulse Rate | x x x x x x x | | | | | | | |
| ECG (12 lead) | x x x | | | | | | | |
| Study Medication  Placebo  Monotherapy  Double-combi.  Triple-combi. | x x x x x  X---------  X--------  X--------X---------X----------  X---------X---------- | | | | | | | |
| Concom Medication | x x x x x x x | | | | | | | |
| Safety & Efficacy Laboratory | x x x | | | | | | | |
| Compliance | x x x x | | | | | | | |
| Adverse Events | x x x x x x x | | | | | | | |
